# Gamma Radiation-Induced Damage in the Zinc Finger of the Transcription Factor IIIA

**DOI:** 10.1155/2016/1642064

**Published:** 2016-10-10

**Authors:** XiaoHong Zhang, YuJi Miao, XiaoDan Hu, Rui Min, PeiDang Liu, HaiQian Zhang

**Affiliations:** ^1^Department of Nuclear Science and Engineering, Nanjing University of Aeronautics and Astronautics, Nanjing 210016, China; ^2^Jiangsu Key Laboratory for Biomaterials and Devices, Southeast University, Nanjing 210009, China; ^3^Collaborative Innovation Center of Radiation Medicine, Jiangsu Higher Education Institutions, Suzhou University, Suzhou 215123, China; ^4^Division of Radiation Medicine, Department of Naval Medicine, Second Military Medical University, Shanghai 200433, China

## Abstract

A zinc finger motif is an element of proteins that can specifically recognize and bind to DNA. Because they contain multiple cysteine residues, zinc finger motifs possess redox properties. Ionizing radiation generates a variety of free radicals in organisms. Zinc finger motifs, therefore, may be a target of ionizing radiation. The effect of gamma radiation on the zinc finger motifs in transcription factor IIIA (TFIIIA), a zinc finger protein, was investigated. TFIIIA was exposed to different gamma doses from ^60^Co sources. The dose rates were 0.20 Gy/min and 800 Gy/h, respectively. The binding capacity of zinc finger motifs in TFIIIA was determined using an electrophoretic mobility shift assay. We found that 1000 Gy of gamma radiation impaired the function of the zinc finger motifs in TFIIIA. The sites of radiation-induced damage in the zinc finger were the thiol groups of cysteine residues and zinc (II) ions. The thiol groups were oxidized to form disulfide bonds and the zinc (II) ions were indicated to be reduced to zinc atoms. These results indicate that the zinc finger motif is a target domain for gamma radiation, which may decrease 5S rRNA expression via impairment of the zinc finger motifs in TFIIIA.

## 1. Introduction

Zinc finger proteins are one of the largest protein families in eukaryotes, and approximately 2-3% of all human genes encode zinc finger proteins [1]. The term “zinc finger” denotes a variety of compact protein domains with a tetrahedral geometry stabilized by structural zinc II ions that interact with cysteine thiol and histidine imidazole groups [2]. The zinc finger motif promotes specific protein-nucleic acid recognition and binding [3], and most zinc finger proteins function as transcription factors. Thus, it can be inferred that agents that damage the zinc finger motif may also impair gene expression [4, 5]. Hence, investigations of factors that damage zinc finger motifs have received much attention in research on zinc finger protein-related regulation of gene expression. Zinc finger motifs can be divided into Cys_8_, Cys_4_, Cys_3_His, and Cys_2_His_2_ types and others. The presence of multiple cysteine residues indicates that zinc finger motifs possess redox properties because most redox reactions in proteins occur at the thiol group of cysteine residues [6]. Based on their redox properties, agents that might impair the activity of zinc finger motifs have been investigated. Several studies have demonstrated that zinc finger motifs are susceptible to* in vitro* oxidation induced by agents such as 3-nitrosobenzamide, hydrogen peroxide, and disulfiram [7–10]. These observations were also confirmed* in vivo*, when repression of a gene specific to Sp-1, a zinc finger protein, was observed in cultured HeLa cells subjected to oxidative stress [11]. The biological effects of ionizing radiation include oxidation or reduction of biological materials. As an indirect effect, many water radiolysis products are produced. Thus, zinc finger motifs are likely targets of ionizing radiation.

A zinc atom is a relatively redox-inert element with an oxidation state of zinc (II). In animal cells, zinc (II) ions are stored in cytosolic cysteine-rich proteins or metallothioneins, in the form of zinc-sulfur clusters. The level of “free” intracellular zinc (II) ions is very low, that is, in the nanopicomolar range, and spontaneous release of zinc (II) ions from the metallothioneins system to target proteins does not occurs [12]. In addition to damaging zinc finger motifs, oxidants may be involved in zinc metabolism. Current investigations suggest that the effects of oxidants on zinc finger motifs are linked to the release of zinc (II) ions [13]. According to this hypothesis, zinc fingers in metalloproteins may serve as a zinc pool. Ionizing radiation differs from oxidative agents that are commonly used for oxidation of zinc finger motifs, because they induce formation of not only the oxidative radical but also the reduced radical, that is, the hydrated electron. The presence of the hydrated electron may reduce zinc II ions.

Transcription factor IIIA (TFIIIA) was the first zinc finger protein to be identified in* Xenopus*, and it consists of nine tandem repeat Cys_2_His_2_-type zinc fingers [14, 15]. The three-dimensional conformation of this type of zinc finger includes an *α*-helix and two *β*-sheets. The internal control region (ICR) of the 5S rRNA gene is a 120-base sequence including three separate promoter regions of an A-box (+50 to +64), an intermediate element (+67 to +72), and a C-box (+82 to +92) [16]. The nine zinc fingers of TFIIIA specifically bind with the ICR through insertion of their *α*-helixes into the major or minor grooves of these three promoter regions [17]. TFIIIA was chosen to investigate damage to the zinc finger motif, because Cys_2_His_2_-type zinc fingers are relatively electrically neutral and possess the low conditional association constant among that of all known zinc finger proteins [18, 19]. In this experiment, we explored the effect of ionizing radiation on the zinc finger motif of TFIIIA and the corresponding mechanism of damage. Specifically, we investigated the binding capacity of the zinc finger in gamma-irradiated TFIIIA with the ICR and identified potential sites of irradiation-induced damage. To the best of our knowledge, only one article has been published on the effects of ionizing radiation on a protein containing zinc-sulfur clusters, that is, metallothionein, with the aim of analyzing its radioprotection function [20]. Our results indicate that gamma radiation affects specific binding of TFIIIA with the ICR through thiol/disulfide and zinc (II) ion/zinc atom exchanges in the zinc finger motifs.

## 2. Materials and Methods

### 2.1. Preparation of Plasmid pMD18T-ICR

The full ICR sequence was synthesized on an automated DNA synthesizer (ABI 394, Applied Biosystems Inc., Carlsbad, CA, USA) using phosphoramidite chemistry. The ICR sequence was then ligated to a pMD18T vector (TaKaRa Co., Dalian, Liaoning, China), which was transformed into* Escherichia coli *competent cells (TaKaRa). The targeted DNA was amplified by polymerase chain reaction (PCR), analyzed by agarose electrophoresis, and confirmed by DNA sequencing.

### 2.2. Preparation of Recombinant TFIIIA

Recombinant* Xenopus laevis* TFIIIA was produced by* Escherichia coli* BL21 (DE3) cells harboring the plasmid pcoldII-TFIIIA [21]. Briefly, cells were cultured at 37°C with shaking at 220 rpm, and when the cell density (OD600) reached 0.6, TFIIIA expression was induced by addition of 0.1 mM isopropylthio-*β*-d-galactoside (IPTG) and 10 *μ*M ZnCl_2_. After 4 h of further incubation at 37°C, cells were harvested and lysed with an EmulsiFlex-C3 homogenizer (Avestin, Ottawa, ON, Canada). After cell lysis, the inclusion bodies, which contain most of the TFIIIA, were first washed with buffer 1 (50 mM Tris-Cl, 100 mM NaCl, 1 mM EDTA, and 0.1% Triton X-100, pH 8.0) before being dissolved in buffer 2 (50 mM Tris-Cl, 100 mM NaCl, 6 M guanidine hydrochloride, and 5 mM DTT, pH 8.0). The whole dissolution process was performed at 4°C, and 24 h was required to completely dissolve the TFIIIA. The supernatant was then slowly diluted in buffer 3 (50 mM Tris-Cl, 50 mM NaCl, 100 *μ*M ZnCl_2_, 0.1 M Arg, 1 mM GSH, 0.1 mM GSSG, 10% glycerol, and 0.1% Nonidet P-40, pH 8.0) at a speed of one drop per second, and the sample was slowly stirred at 4°C for 24 h to refold the TFIIIA. After the refolding process was complete, the sample was concentrated using an ultrafiltration tube (Amicon Ultra, Millipore Co., Billerica, MA, USA) before being purified by fast protein liquid chromatography using a Superdex 75 column (GE Co., Fairfield, CT, USA).

To detect zinc (II) ions in irradiated TFIIIA, recombinant TFIIIA was also prepared in buffer without zinc (II) ion supplementation. Similar to the procedure described above, the TFIIIA contained in inclusion bodies was refolded using buffers 1, 2, and 3. The refolded TFIIIA was then precipitated with 40% ammonium sulfate at 4°C for 30 min. The pellet was again denatured with buffer 2 and refolded in buffer 3 lacking the 100 *μ*M ZnCl_2_. The rest of the purification process was performed as described above. The protein concentration was measured using the Bradford method, and the purified TFIIIA solution (0.25 *μ*g/*μ*L) was stored at −80°C until use.

### 2.3. Radiation Exposure

The TFIIIA solution was irradiated with ^60^Co sources in an atmosphere of air at 4°C. The absorbed doses were 0 Gy, 1 Gy, 10 Gy, 100 Gy, 500 Gy, 800 Gy, and 1000 Gy. The low doses (1 Gy and 10 Gy) were administered with ^60^Co source (Gaotong Isotope Co., Chengdu, Sichuan, China) with a radioactivity of approximately 22.2 × 10^12^ Bq. The dose rate was 0.20 Gy/min. The high doses (100 Gy, 500 Gy, 800 Gy, and 1000 Gy) were administered with ^60^Co source (Nordion Inc., Kanata, ON, Canada) with a radioactivity of approximately 14.8 × 10^16^ Bq. The dose rate was 800 Gy/h. Dosimetry was performed on a regular basis with a 0.6 cm^3^ Farmer Ionization Chamber (Type 30010) which was connected to a dosimeter (PTW-Freiburg Co., Freiburg, Breisgau, Germany). The chamber was placed next to the tubes for irradiation.

### 2.4. Electrophoretic Mobility Shift Assay

For preparation of fluorescent FAM-labeled probes, the ICR of the 5S rRNA gene was amplified by PCR using a high-fidelity Dpx DNA Polymerase (Tolo Biotech., Shanghai, China) from the plasmid pMD18T-ICR using the primers M13F-47(FAM) and M13R-48. The FAM-labeled probes were purified using the Wizard SV Gel and PCR Clean-Up System (Promega Co., Madison, WI, USA) and were quantified with a NanoDrop 2000c System (Thermo Co., Waltham, MA, USA). Electrophoretic mobility shift assay (EMSA) was performed in a 20 *μ*L reaction volume containing 50 ng of probe and 2.5 *μ*g of irradiated TFIIIA, in a reaction buffer of 50 mM Tris-HCl (pH 8.0), 100 mM KCl, 2.5 mM MgCl_2_, 0.05 mM DTT, and 10% glycerol. After incubation for 30 min at 25°C, the reaction system was loaded onto a 2% agarose gel buffered with 0.5 × TBE. Gels were scanned with an ImageQuant LAS 4000 mini system (GE). Sheared salmon sperm DNA (100 ng/*μ*L) was added to prevent nonspecific binding.

### 2.5. Quantification of Disulfide in Irradiated TFIIIA

The procedures for disulfide detection were as follows: TFIIIA solutions (0.25 *μ*g/*μ*L, 200 *μ*L) were pipetted into the 5,5′-dithiobis-2-nitrobenzoic acid (DTNB) and 2-nitro-5-thiosulfobenzoate (NTSB) solution (3 mL), respectively. The DTNB solution (pH 8.0) was prepared with 8 M urea, 200 mM Tris, 1% SDS, 3 mM EDTA, and 10 mM DTNB. The NTSB solution was prepared from the stock solution (100 mg of DTNB was dissolved in 10 mL of 1 M Na_2_SO_3_, with the pH adjusted to 7.5; the bright red solution was brought to 38°C, and oxygen was bubbled through it) by diluting it 1 : 100 with a freshly prepared solution containing 8 M urea, 200 mM Tris, 100 mM sodium sulfite, 1% SDS, and 3 mM EDTA. The mixtures of protein and DTNB/NTSB solution were incubated in the dark for 30 min. The absorbance at 412 nm was then recorded against a blank of 3 mL of DTNB or NTSB solution and 200 *μ*L of water. The absorbance at 412 nm for the DTNB mixture was found to increase linearly with free thiol contents and that for the NTSB mixture with total thiol contents. Thus, the concentration of disulfide can be acquired by using the following formula:

(1) Disulfidemmol/g=A412NTSB×D×10−6C×V×ε−A412DTNB×D×10−6C×V×ε,

where *D* is the dilution multiple, *C* is the concentration of TFIIIA, *V* is the volume of TFIIIA, and *ɛ* is the extinction coefficient (13900 M^−1^ cm^−1^).

### 2.6. Quantification of Zinc (II) Ions in Irradiated TFIIIA

A zinc (II) ion assay kit (Jiancheng Co., Nanjing, Jiangsu, China), which is based on a colorimetric method, was used to determine the concentration of zinc (II) ions in gamma-irradiated TFIIIA. The following procedures were used: Reagent 1 (ascorbic acid and sodium citrate dissolved in HEPES buffer (0.2 M, pH 6.0), with final concentrations of 0.05 M ascorbic acid and 0.2 M sodium citrate) was mixed with irradiated TFIIIA (150 *μ*L) and the standard solution (150 *μ*L, 30.6 *μ*M; ZnSO_4_). The solution was incubated for 5 min at 37°C. The optical density of the solution was then read as *A*1 at 578 nm using an ultraviolet-visible spectrophotometer (Shimadzu Co., Kyoto, Japan). After the reading, 600 *μ*L of 5-Br-PAPS was added to the solution. The mixed solution was incubated for 5 min at 37°C. The optical density of the solution was read as *A*2. The concentration of zinc (II) ions was calculated using the following formula:

(2) $$\begin{array}{c} C_{Zn^{2+}}\left(\;\mu mol/L\;\right)=C_{standard}\times \frac{A2_{sample}-A1_{sample}}{A2_{standard}-A1_{standard}}. \end{array}$$

## 3. Results and Discussion

### 3.1. Binding of Gamma-Irradiated TFIIIA with the ICR

**Figure 1:**
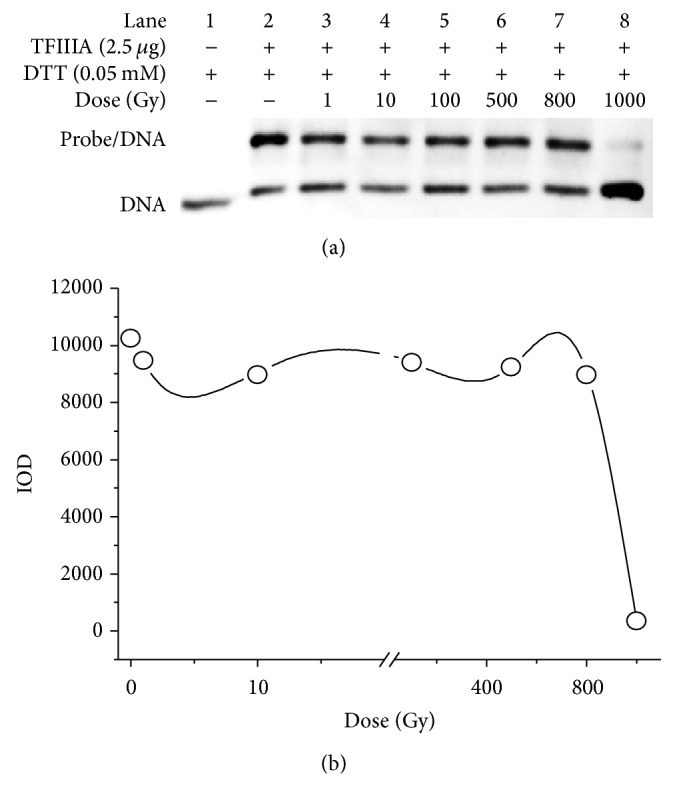
Binding of irradiated transcription factor IIIA (TFIIIA) with the internal control region (ICR). (a) Band of binding of gamma-irradiated TFIIIA (0.25 *μ*g/*μ*L; 10 *µ*L) with the ICR (the final concentration 2.5 ng/*µ*L). Gamma irradiation was administered at doses of 1 Gy, 10 Gy, 100 Gy, 500 Gy, 800 Gy, and 1000 Gy. (b) Integral optical density (IOD) of the binding.

The effect of gamma radiation on the binding of TFIIIA with the ICR was studied using EMSA. Different doses of gamma rays, ranging from 1 Gy to 1000 Gy, were used to irradiate the TFIIIA solution.  Figure 1(a) shows the EMSA band for binding of irradiated TFIIIA with the ICR, and Figure 1(b) shows the integral optical density (IOD) of each band. As shown in Figure 1(a), gamma doses equal to or below 800 Gy exerted no significant effect on the binding of TFIIIA with the ICR, and TFIIIA irradiated with these doses could still bind with the ICR (lanes 3–7). The results are concordant with the corresponding IODs (Figure 1(b)). However, 1000 Gy of gamma irradiation eliminated this binding capacity, and, at this dose, binding of TFIIIA with the ICR was significantly decreased (lane 8). The corresponding IOD for 1000 Gy was 345.70. These results indicate that 1000 Gy of gamma radiation impairs the function of zinc finger motifs in TFIIIA and decreases specific binding of TFIIIA with the ICR. Binding of TFIIIA with the ICR is the first step in expression of 5S rRNA; the decrease in the specific binding of TFIIIA with the ICR may therefore affect the expression of 5S rRNA.

### 3.2. Damage Sites in the Zinc Finger Motif of Gamma-Irradiated TFIIIA

**Figure 2:**
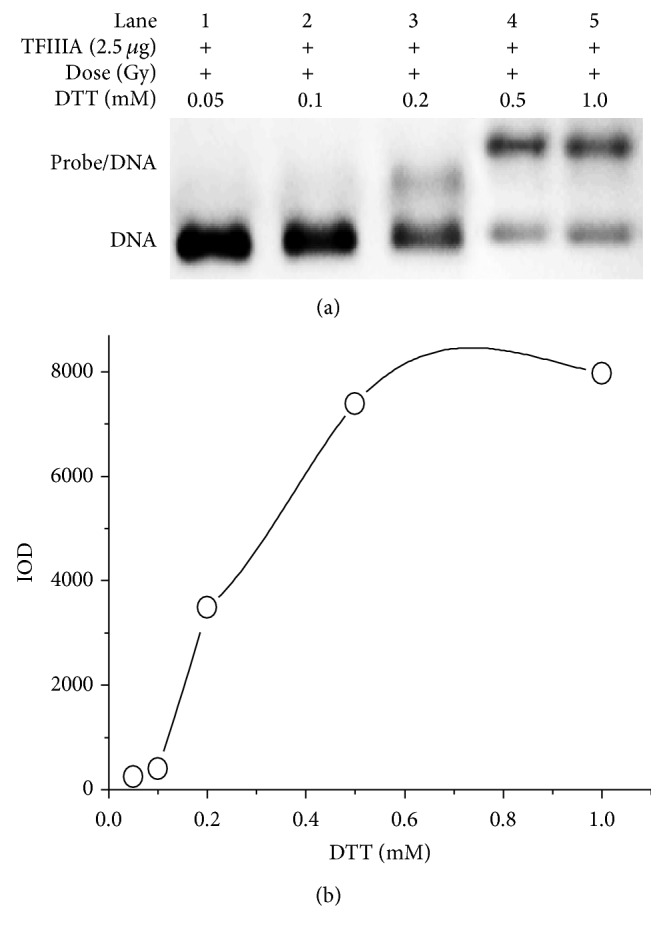
Restoration of the binding capacity of irradiated transcription factor IIIA (TFIIIA) with the internal control region (ICR) by DL-dithiothreitol (DTT) solution. (a) Band of binding of gamma-irradiated TFIIIA (1000 Gy) with the ICR after irradiated TFIIIA was incubated with different concentrations of DTT (0.05 mM, 0.1 mM, 0.2 mM, 0.5 mM, and 1.0 mM). (b) Integral optical density (IOD) of the corresponding binding.

The redox potential of the sulfur atom in proteins ranges from −0.27 to −0.125 V [22]. The low redox potential indicates that the thiol group in zinc finger motifs is readily oxidized. Gamma radiation-induced impairment of the binding capacity of TFIIIA might involve oxidation of the thiol groups in zinc finger motifs. dl-dithiothreitol (DTT), an agent that reduces the reversibly oxidative thiol group, was used to explore whether the thiol groups in the zinc finger motifs of irradiated TFIIIA were oxidized.  Figure 2(a) presents the EMSA bands for binding of irradiated TFIIIA with the ICR after irradiated TFIIIA was incubated with different concentrations of DTT, and Figure 2(b) shows the IOD of each band. The concentrations of DTT used were 0.05 mM, 0.1 mM, 0.2 mM, 0.5 mM, and 1.0 mM, respectively. The radiation dose was 1000 Gy. As shown in Figure 2(a), DTT at 0.1 mM resulted in no significant recovery in the binding capacity of irradiated TFIIIA (lane 2). The corresponding IOD was only 398.44 (Figure 2(b)). Application of DTT at 0.2 mM produced a slight recovery (lane 3). However, the recovery induced by DTT at 0.5 mM was significant (lane 4), with IOD of 7378.14. Furthermore, application of DTT at 1.0 mM produced a similar recovery to that induced by 0.5 mM of DTT (lane 5), with IOD of 7963.55. The products of oxidation of the thiol group can be reversible or irreversible. The reversible end product is disulfide [23], and the irreversible products are sulfinic acid and sulfonic acid. The recovery of zinc finger binding capacity observed following incubation of irradiated TFIIIA with DTT indicates that the water radiolysis products of gamma radiation oxidize the thiol groups of zinc finger motifs to generate disulfide products.

**Figure 3:**
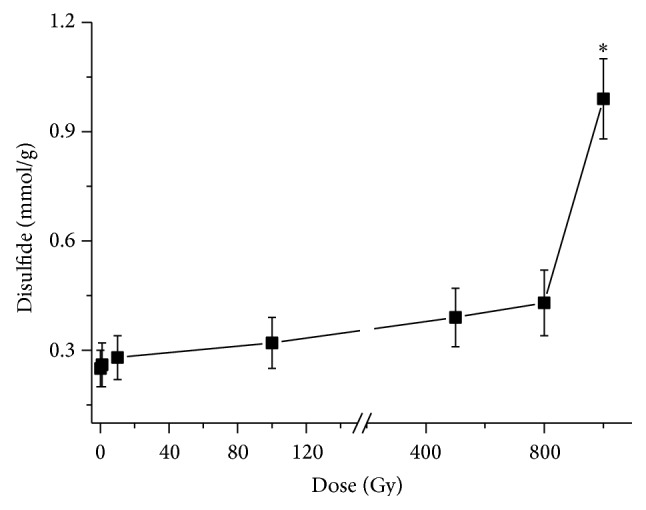
Concentration of disulfide in irradiated transcription factor IIIA (TFIIIA). Gamma irradiation was administered at doses of 1 Gy, 10 Gy, 100 Gy, 500 Gy, 800 Gy, and 1000 Gy. Asterisks represent the significance level compared to the control group (0 Gy) (^*∗*^ *P* < 0.05).

In order to further demonstrate the result obtained in Figure 2, the concentration of disulfide in irradiated TFIIIA was detected with the use of the colorimetric method. The gamma doses used were from 1 Gy to 1000 Gy.  Figure 3 shows the concentrations of disulfide in TFIIIA irradiated with different gamma doses. The concentration of disulfide increases with increasing dose, and disulfide in 1000 Gy irradiated TFIIIA is significantly higher than that in the control group. The result supports the indication that the water radiolysis products of 1000 Gy of gamma radiation oxidize the thiol groups of zinc finger motifs to form the disulfide products. The water radiolysis products are hydroxyl free radical, hydrogen peroxide, hydrated electron, and hydrogen free radical. Their *G* values are 2.7, 0.55, 2.7, and 0.7, respectively. The corresponding reactions of formation of disulfide are as follows:

(3) NH2–CH(COOH)–CH2–SH+OH•⟶NH2–CH(COOH)–CH2–S•+H2ONH2–CH(COOH)–CH2–SH+eaq−⟶NH2–CH(COOH)–CH3•+SH•NH2–CH(COOH)–CH2–SH+H•⟶NH2–CH(COOH)–CH2–S•+H2NH2–CH(COOH)–CH2–SH+H•⟶NH2–CH(COOH)–CH3•+H2SNH2–CH(COOH)–CH2–SH+NH2–CH(COOH)–CH3•⟶NH2–CH(COOH)–CH2–S•+CH3CH(NH2)COOH2NH2–CH(COOH)–CH2–S•⟶NH2–CH(COOH)–CH2–S–S–CH2–CH(COOH)–NH22NH2–CH(COOH)–CH2–SH+H2O2⟶NH2–CH(COOH)–CH2–S–S–CH2–CH(COOH)–NH2+2H2O

Several studies have demonstrated that formation of the disulfide bond in zinc finger motif leads directly to release of the zinc (II) ion [13, 24]. This release promotes final collapse of the structure of the zinc finger motif, which may be the reason for the decrease in the binding of irradiated TFIIIA with ICR.

**Figure 4:**
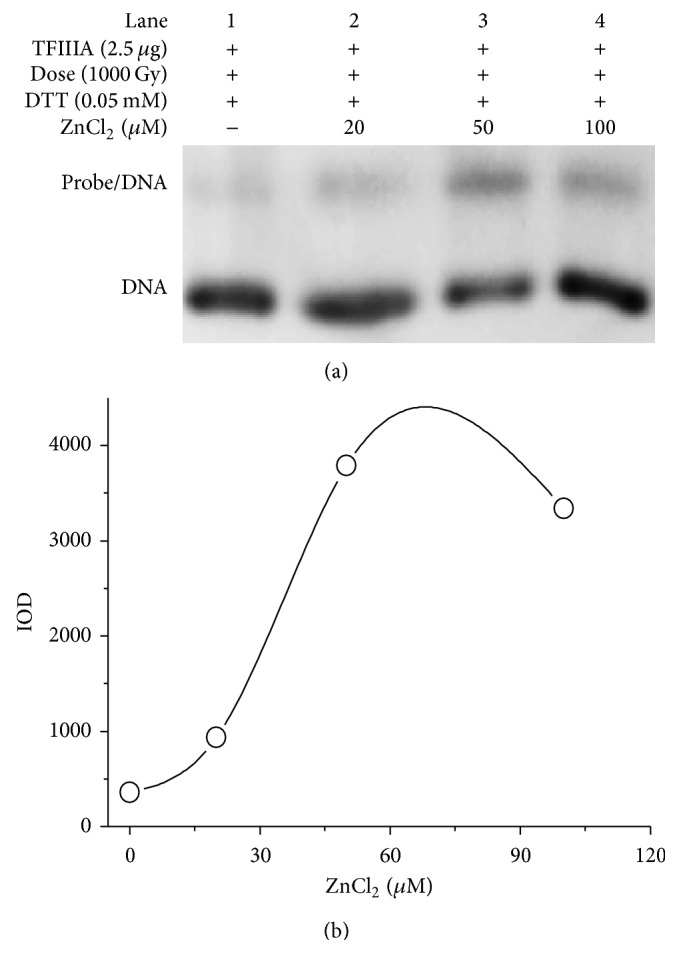
Restoration of the binding capacity of irradiated transcription factor IIIA (TFIIIA) with the internal control region (ICR) by ZnCl_2_ solution. (a) Band of binding of gamma-irradiated TFIIIA (1000 Gy) with the ICR after irradiated TFIIIA was incubated with different concentrations of ZnCl_2_ (20 *µ*M, 50 *µ*M, and 100 *µ*M). (b) Integral optical density (IOD) of the corresponding binding.

Although binding of TFIIIA with the ICR was affected by thiol/disulfide exchange, the modulation was partial because DTT at 0.5 or 1.0 mM did not fully recover the binding capacity of irradiated TFIIIA (Figure 2(a), lanes 4 and 5). The cysteine residues and zinc ions are essential determinants of the structure of various types of zinc finger motifs, such as Cys_8_ and Cys_4_, which indicates that the thiol group and the zinc binding site are very important for the structure and function of the zinc finger. The finding that DTT partially restored the binding capacity of irradiated TFIIIA suggests that the zinc site, in addition to the thiol group, might be involved in the damaging of zinc finger motifs.  Figure 4(a) presents the EMSA band results for binding of irradiated TFIIIA with the ICR after irradiated TFIIIA was incubated with different concentrations of ZnCl_2_.  Figure 4(b) shows the IOD of each band. The concentrations of ZnCl_2_ used were 20 *μ*M, 50 *μ*M, and 100 *μ*M, respectively. As shown in Figure 4(a), ZnCl_2_ at 20 *μ*M produced a slight recovery of the binding capacity of irradiated TFIIIA (lane 2). The corresponding IOD was 929.92. However, ZnCl_2_ at 50 *μ*M resulted in a relatively significant recovery of the binding capacity (lane 3), and the IOD was 3784.67. Treatment with 100 *μ*M ZnCl_2_ produced IOD of 3338.12 (lane 4 and Figure 4(b)). These results suggest that the zinc site is involved in gamma radiation-induced damage in zinc finger motifs.

**Figure 5:**
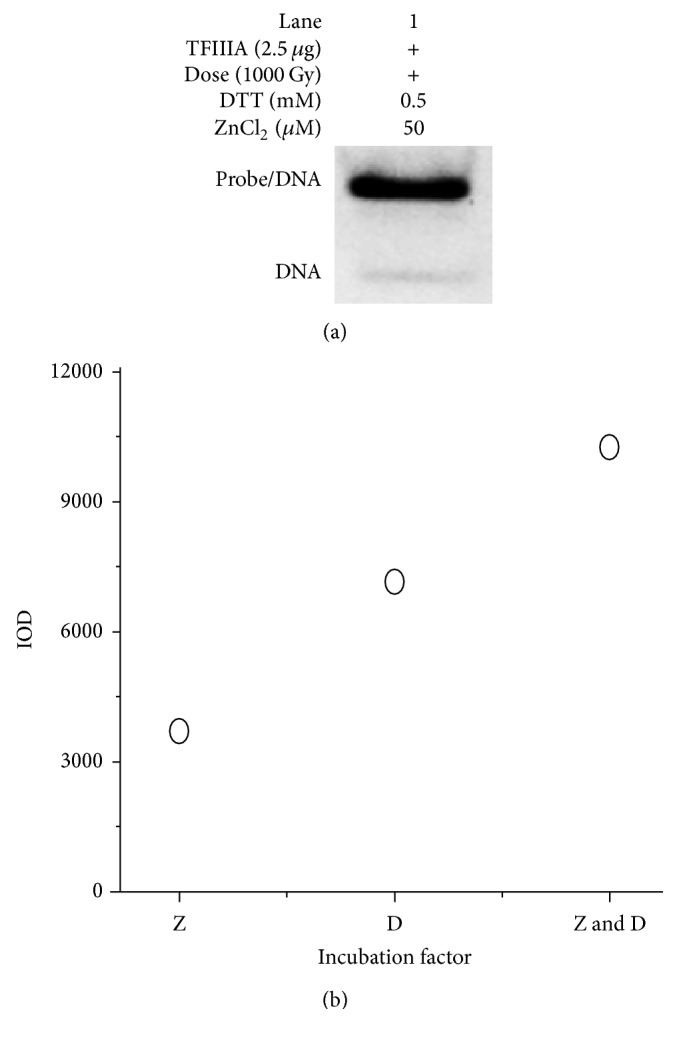
Restoration of the binding capacity of irradiated transcription factor IIIA (TFIIIA) with the internal control region (ICR) by DL-dithiothreitol (DTT) and ZnCl_2_ solutions. (a) Band of binding of gamma-irradiated TFIIIA (1000 Gy) with the ICR after irradiated TFIIIA was incubated with 0.5 mM of DTT and 50 *µ*M of ZnCl_2_. (b) Integral optical density (IOD) of the binding after irradiated TFIIIA was incubated with ZnCl_2_ (50 *µ*M), DTT (0.5 mM), and DTT (0.5 mM) and ZnCl_2_ (50 *µ*M), respectively. Z: 50 *µ*M of ZnCl_2_. D: 0.5 mM of DTT.

The binding capacity of irradiated TFIIIA was fully restored after it was incubated with both 50 *μ*M ZnCl_2_ and 0.5 mM DTT (Figure 5(a)). The corresponding IOD was 10156.85 (Figure 5(b)). The full recovery produced by the combination of these two agents further demonstrates that damage to the zinc finger motifs in TFIIIA by 1000 Gy of gamma radiation involves both the thiol group and the zinc site.

### 3.3. Damage to the Zinc Site of Zinc Finger Motifs in Gamma-Irradiated TFIIIA

Although the thiol group reduced by DTT could recapture the existing zinc (II) ion to reconstruct the function of the zinc finger, a full recovery of function only occurred after the reduced irradiated TFIIIA was incubated with a fresh ZnCl_2_ solution (Figure 5(a)). This result suggests that some of the zinc (II) ions in irradiated TFIIIA are altered. As zinc is a metal ion, such an alteration would occur in the form of changed valence state. To date, no method has been developed to directly detect changes in valence induced by ionizing radiation in metal ions in solution. The colorimetric method for determination of the concentration of the zinc (II) ion was therefore used to indirectly assess whether the valence of the zinc ion changed in irradiated TFIIIA. The buffer of the irradiated TFIIIA did not contain zinc ions.  Table 1 shows the content of zinc (II) ions in TFIIIA irradiated with 1000 Gy; the concentration of zinc (II) ions was lower in irradiated TFIIIA than in nonirradiated TFIIIA. The valence of zinc atoms was 0 and II, respectively.

The decrease in the concentration of zinc (II) ions indirectly indicates that zinc (II) ions underwent reduction, and zinc atoms were generated in irradiated TFIIIA. Gamma radiation-induced hydrated electrons are a strong reduction agent with a redox potential of 2.77 V. The zinc atoms in irradiated TFIIIA were generated by hydrated electrons. The corresponding reaction is as follows:

(4) $$\begin{array}{c} {Zn}^{2+}+2e_{aq}^{-}⟶Zn \end{array}$$

Organisms protect themselves against metal elements by using various proteins to sequester the metal elements. Generated zinc atoms function as free radicals and might be an important source of radiation damage. Importantly, the formation of zinc atoms in irradiated TFIIIA suggests that valence electrons outside of the atomic nuclei of metal ions are susceptible to ionizing radiation. Valence electrons outside of the atomic nuclei of metal ions may be a new radiation sensitive site. Metalloproteins are involved in various physiological processes, and their susceptibility to radiation effects based on this sensitive site, therefore, is significant in radiation biology.

## 4. Conclusion

TFIIIA is a transcription factor that specifically binds to the 5S rRNA gene through formation of zinc finger-ICR complexes. Gamma radiation (1000 Gy) can impair binding of zinc finger motifs with the ICR. The sites of radiation-induced damage in the zinc finger motif are the thiol groups of the cysteine residues and zinc (II) ions. The thiol groups were oxidized to form disulfide bonds and the zinc (II) ions may be reduced to zinc atoms. In this experiment, we obtained* in vitro* data on gamma radiation-induced damage to the zinc finger motifs of TFIIIA. Further work should focus on gamma radiation-induced impairment of the zinc finger motif* in vivo*. The effects of radiation on proteins containing metal ions and/or thiol groups are significant and merit further investigation.

## Figures and Tables

**Table 1:** Contents of the zinc (II) ion ($\overset{-}{x}\pm SD$) in gamma-irradiated TFIIIA (*n* = 4).

Dose (Gy)	Zinc (II) ion (*μ*mol/L)
0	18.72 ± 0.28
1000	12.15 ± 1.49^*∗*^

^*∗*^Compared to 0 Gy group, *P* < 0.05.
